# Nitric Oxide-Releasing Nanoparticles Are Similar to Efinaconazole in Their Capacity to Eradicate *Trichophyton rubrum* Biofilms

**DOI:** 10.3389/fcimb.2021.684150

**Published:** 2021-07-15

**Authors:** Caroline Barcelos Costa-Orlandi, Luis R. Martinez, Níura Madalena Bila, Joel M. Friedman, Adam J. Friedman, Maria José S. Mendes-Giannini, Joshua D. Nosanchuk

**Affiliations:** ^1^ Department of Medicine, Division of Infectious Diseases, Albert Einstein College of Medicine, Bronx, NY, United States; ^2^ Deparment of Clinical Analysis, School of Pharmaceutical Sciences, Sao Paulo State University (UNESP), Araraquara, Brazil; ^3^ Department of Oral Biology, College of Dentistry, University of Florida, Gainesville, FL, United States; ^4^ Department of Para-Clinic, School of Veterinary, Universidade Eduardo Mondlane (UEM), Maputo, Mozambique; ^5^ Department of Physiology and Biophysics, Albert Einstein College of Medicine, Bronx, NY, United States; ^6^ Department of Dermatology, George Washington School of Medicine and Health Sciences, Washington, DC, United States; ^7^ Department of Medicine, Division of Dermatology, Albert Einstein College of Medicine, Bronx, NY, United States; ^8^ Department of Microbiology and Immunology, Albert Einstein College of Medicine, Bronx, NY, United States

**Keywords:** *Trichophyton rubrum*, biofilms, nitric oxide, nanoparticles, antifungal drugs, efinaconazole

## Abstract

Filamentous fungi such as *Trichophyton rubrum* and *T. mentagrophytes*, the main causative agents of onychomycosis, have been recognized as biofilm-forming microorganisms. Nitric oxide-releasing nanoparticles (NO-np) are currently in development for the management of superficial and deep bacterial and fungal infections, with documented activity against biofilms. In this context, this work aimed to evaluate, for the first time, the *in vitro* anti-*T. rubrum* biofilm potential of NO-np using standard ATCC MYA-4438 and clinical BR1A strains and compare it to commonly used antifungal drugs including fluconazole, terbinafine and efinaconazole. The biofilms formed by the standard strain produced more biomass than those from the clinical strain. NO-np, fluconazole, terbinafine, and efinaconazole inhibited the *in vitro* growth of planktonic *T. rubrum* cells. Similarly, NO-np reduced the metabolic activities of clinical strain BR1A preformed biofilms at the highest concentration tested (SMIC_50_ = 40 mg/mL). Scanning electron and confocal microscopy revealed that NO-np and efinaconazole severely damaged established biofilms for both strains, resulting in collapse of hyphal cell walls and reduced the density, extracellular matrix and thickness of the biofilms. These findings suggest that biofilms should be considered when developing and testing new drugs for the treatment of dermatophytosis. Development of a biofilm phenotype by these fungi may explain the resistance of dermatophytes to some antifungals and why prolonged treatment is usually required for onychomycosis.

## Introduction

Dermatophytoses, infections caused by dermatophytes, are considered the most prevalent mycoses worldwide, affecting ~20 to 25% of the population, regardless of the demographic examined (Havlickova et al., 2008; Zhan and Liu, 2017; Costa-Orlandi et al., 2020). While typically involving the stratum corneum in healthy individuals, follicular and dermal invasion can occur in various clinical scenarios, such as immunosuppression, and can be a portal for polymicrobial infections (de Sousa Mda et al., 2015; Gupta et al., 2017; Pereira, 2021). The ability of these fungi to form biofilms has been widely studied in recent years (Costa-Orlandi et al., 2014; Brilhante et al., 2017; Costa-Orlandi et al., 2017; Danielli et al., 2017; Chen et al., 2019; Costa-Orlandi et al., 2020; Garcia et al., 2020; Bila et al., 2021). Biofilm formation by dermatophytes, especially in the setting of onychomycosis (Burkhart et al., 2002; Gupta et al., 2016; Lipner and Scher, 2019; Gupta and Foley, 2019), further complicates and limits current treatment strategies, as they are highly resistant to antimicrobials (Ramage et al., 2012). Moreover, the treatment of onychomycosis is protracted, typically months, and resolution is often incomplete, requiring repeat treatment courses (Gupta et al., 2016; Gupta and Foley, 2019). A major factor in the poor efficacy of antifungal drugs in the treatment of onychomycosis is their low penetration in nails, whether administered topical or systemic (Iwanaga et al., 2017; Costa-Orlandi et al., 2018).

Given that biofilms formed by dermatophytes are resistant to conventional antifungals (Brilhante et al., 2018; Costa-Orlandi et al., 2020; Garcia et al., 2020) and the established difficulty in and cost of treating onychomycosis, new approaches to overcome these impediments are urgently needed. Nitric oxide (NO) is an endogenous, diatomic, and simple molecule that has important physiological functions, including important roles in the body’s defenses to infections (Friedman et al., 2008; Schairer et al., 2012b). It is an unstable gas that, in an aerobic environment, reacts with oxygen (O_2_) and/or superoxide radicals. These reactions result in the formation of reactive nitrogen oxide species (RNOS) and oxygen intermediates (ROS) that cause nitrosative and oxidative damage through DNA alteration, enzyme inhibition and induction of lipid peroxidation, which are responsible for most of the antimicrobial properties of NO (Schairer et al., 2012b). Because they are lipophilic, NO molecules easily cross physiological barriers and, consequently, reach most target cells. Therefore, NO’s target must be close to the place where the molecule is generated (Friedman et al., 2008). The antimicrobial effect is exerted in two ways that are dependent on concentration. At low concentrations (≤1 µM), NO acts as a signaling molecule promoting the growth and activity of cells in the immune system, and, at high concentrations (>1 µM), NO molecules covalently bind to macromolecules, inhibiting or killing target pathogens (Schairer et al., 2012b).

Several NO delivery systems have been developed to provide the delivery of this molecule in a safe, effective and convenient way (Martinez et al., 2009; Han et al., 2012; Schairer et al., 2012a; Schairer et al., 2012b; Cabral et al., 2019; Pieretti et al., 2021). Nanoscale particles (1-100 nm) can create a depot effect on the surface of the skin and nails owing to the increase resident time in the local milieu, providing long-lasting treatment per application. In addition, NO nanoparticles (NO-np) offer greater skin penetration, increasing the probability of interaction with fungal cells (Han et al., 2011; Mordorski et al., 2017).

We developed a stable and low-cost NO generating/releasing platform, using nanotechnology based on a silane hydrogel (Friedman et al., 2008). The antimicrobial activity of these NO-np against a wide-range of Gram positive and negative bacteria (Friedman et al, 2011) has been consistently demonstrated. In addition to bacteria, these NO-np were also effective against diverse species of fungi including *Candida albicans* (Macherla et al., 2012) and *Trichophyton* species (Mordorski et al., 2017; Costa-Orlandi et al., 2018). These NO-np have also been tested *in vivo* on wounds caused by microorganisms and a decrease in inflammation and microbial loads has been shown, resulting in rapid healing (Martinez et al., 2009; Sanchez et al., 2012; Mordorski et al., 2017). The current work aimed to evaluate the *in vitro* anti-*T. rubrum* biofilm potential of NO-np and compare their antifungal activity to conventional antifungal drugs such as terbinafine (TRB), fluconazole (FCZ) and efinaconazole (EFCZ).

## Material And Methods

### Fungi

In this work, strains of *T. rubrum* ATCC MYA-4438, kindly provided by Dr. Mahmoud Ghannoum, from Case Western Reserve University in Cleveland, Ohio, USA and a *T. rubrum* BR1A clinical strain from Montefiore Medical Center, Bronx, NY, USA were used. All strains were maintained on Sabouraud dextrose agar (BD Difco) supplemented with 0.1% chloramphenicol (*Sigma-Aldrich*) and incubated at 28°C (Costa-Orlandi et al., 2012; Costa-Orlandi et al., 2014).

### Synthesis of NO-np

The np were synthesized as previously described (Friedman et al., 2008). Briefly, a silane hydrogel compound was synthesized using a mixture of tetramethylortosilane (*Sigma-Aldrich*), 1mM hydrochloric acid (*Sigma-Aldrich*), polyethylene glycol 400 (*Sigma-Aldrich*), chitosan (*Sigma-Aldrich*), glucose (*Sigma-Aldrich*), and sodium nitrite (*Sigma-Aldrich*) in 50 mM sodium phosphate buffer (*Sigma-Aldrich*) (pH = 7). Nitrite was reduced within the matrix with electrons generated thermally from glucose. After the redox reaction, the reagents were combined and dried on a lyophilizer, resulting in a fine powder. As a negative control, nanoparticles without NO (np) were also synthesized (Friedman et al., 2008; Han et al., 2009; Martinez et al., 2009; Macherla et al., 2012).

### *In Vitro* Characterization of Biofilm Formation

To verify the ability to form biofilms, both strains of *T. rubrum* were subcultured on potato dextrose agar (BD Difco ™) and incubated at 28°C for 7 days. The fungal suspensions were prepared and adjusted to reach a final concentration of 10^6^ conidia/mL. Then, 200 and 1000 μL of the suspensions were added to 96 and 24-well plates, respectively. The plates were incubated at 37°C until pre-adhesion of the biofilms. After this period, the supernatant was gently removed from each well and the plates were washed with sterile saline to remove non-adherent cells. Finally, 200 and 1000 μL of RPMI-1640 medium with L-glutamine, phenol red as a pH indicator (*Sigma-Aldrich*), buffered with MOPS (*Sigma-Aldrich*), and pH = 7 (*Sigma-Aldrich*) were added to the wells of the 96 and 24 well plates. The plates were again incubated at 37°C for up to 96 h in an orbital shaker incubator set at 150 rpm (Costa-Orlandi et al., 2014).

#### Determination of the Metabolic Activity of Biofilms by the 2,3-bis (2-Methoxy-4-Nitro-5-Sulfophenyl) -5- [Carbonyl (Phenylamino)] - 2H-Tetrazolium Hydroxide (XTT) Reduction Assay

The metabolic activities of biofilms were verified using the XTT reduction assay (*Sigma-Aldrich*) at different time intervals (3, 12, 24, 48, 72, and 96 h). XTT stock solutions (1 mg of salt/ml of PBS) and menadione (1 mM in acetone) (*Sigma-Aldrich*) were prepared. Then, 50 µL of XTT and 4 µL of menadione were added to each well and 96-well microtiter plates were incubated at 37°C for 3 h. The colorimetric change was measured in a microtiter plate reader (LabSystems Multiskan MS; LabSystems, Finland), at an optical density of 490 nm. In all experiments, RPMI-1640 medium was included as a negative control (Martinez and Casadevall, 2006).

#### Quantification of Biofilm Biomass by Crystal Violet Staining

For the quantification of biomass, biofilms were formed in 96-well plates, until maturation as previously established above. Then, the medium was removed from each well and the adhered cells were washed three times with sterile 0.85% saline. After drying at RT, 100 µL of 0.5% crystal violet solution (*Sigma-Aldrich*) was added to each well for 5 min. The wells were then washed with sterile distilled water until the excess staining was removed and the biofilms were discolored by adding 100 µL of 95% ethanol to each well. The ethanol was then gently pipetted until the crystal violet was completely solubilized. Finally, the supernatants from each well were transferred to a new 96-well plate and read in a microtiter plate reader (LabSystems Multiskan MS; LabSystems, Finland), at a wavelength of 570 nm (Mowat et al., 2007; Costa-Orlandi et al., 2014).

### Effect of NO-np and Antifungal Drugs on Planktonic Cells and Mature *T. rubrum* Biofilms

To evaluate the effects of NO-np and antifungal drugs (FCZ; TRB; and EFCZ) on planktonic cells and mature biofilms of *T. rubrum*, working solutions were tested in the following concentrations: NO-np: 40 - 0.075 mg/mL; FCZ: 0.512-0.001 mg/mL; TRB: 0.032-0.00006 mg/mL; EFCZ: 0.320 - 0.00006 mg/mL. FCZ (Pfizer) and TRB (*Sigma-Aldrich*) were prepared according to the recommendations in the document M38-A2, proposed by the Clinical and Laboratory Standards Institute (CLSI, 2008). Briefly, stock solutions were prepared and working solutions were diluted in RPMI-1640. For EFCZ (Jublia; Valeant Pharmaceuticals International, Inc.), the working solutions were prepared from the 10% stock. NO-np and np were weighed aseptically, diluted directly in the RPMI-1640 medium and solubilized with the aid of a rod sonicator (10 sec) and vortexed for 2 min.

For planktonic cells, inocula were prepared in the same concentration used for biofilm formation (10^6^ conidia/mL) in RPMI-1640 medium. One hundred µL of the fungal suspensions were co-incubated with the different concentrations of the working solutions of the nanoparticles and drugs. Biofilms were formed in 96-well plates, as described. After maturation, the culture medium was carefully aspirated, and biofilms were washed 3 times with 200 μL of sterile 0.85% saline to remove remaining planktonic cells. Then, the working solutions of NO-np, np, or antifungal drugs were distributed on the plates, with their respective controls. The plates were incubated in an orbital shaker incubator at 150 rpm and 37°C for 72 h. Metabolic activities were assessed by the XTT reduction assay, as described (Pierce et al., 2008; Costa-Orlandi et al., 2020). Biofilms treated with np or drugs were compared to untreated controls to determine differences in metabolic activity reduction.

### Scanning Electron Microscopy (SEM)

To better visualize the damage caused by NO-np and antifungal drugs to biofilms, SEM was performed. Biofilms were formed in 24-well plates. After maturation, they were treated for 72 h with working solutions of the drugs (FCZ: 0.512 mg/mL; TRB: 0.032 mg/mL; EFCZ: 0.320 mg/mL) and NO-np (40 mg/mL). After incubation, the wells were washed with sterile 0.85% saline and the biofilms were fixed with 800 μL of 2.5% glutaraldehyde solution for 1 h at RT. The samples were once again washed and dehydrated with increasing concentrations of ethanol, from 50% to 100%. The bottoms of the plates were cut with a scalpel and dried in a desiccator. Immediately after drying, they were fixed on carbon tape, mounted on aluminum cylinders with silver (stubs) and placed on a high vacuum evaporator for gold coating. The topographies of the biofilms were analyzed using a SEM Jeol JSM-6610LV (Martinez et al., 2010; Costa-Orlandi et al., 2014).

### Confocal Laser Scanning Microscopy

Biofilms were formed in 24-well plates containing previously sterilized round coverslips. After maturation (72 h), the culture medium was carefully aspirated, and the biofilms were treated for 72h with working solutions of EFCZ (0.320 mg/mL) and NO-np (40 mg/mL). The supernatant was removed, the biofilms washed and 100 µL of a PBS solution containing 25 µM ConA (Concanavalin A - conjugated to Alexa fluor 488 - *Invitrogen*) and 10 µM FUN-1 (*Invitrogen*). The plates were incubated at 37°C, for 45 min, protected from light. Then, the coverslips were washed with PBS, removed from the wells and poured under 4 µL of Fluoromount-G (*Sigma-Aldrich*), previously deposited on microscope slides for observation in a Leica TCS SP5 inverted confocal microscope (*Leica Microsystems*) (Martinez and Casadevall, 2006).

### Statistical Analysis

All data were subjected to statistical analysis using GraphPad Prism 9.0 (*GraphPad Software*). *P* values for multiple comparisons were calculated by one-way analysis of variance (ANOVA) and adjusted using the Bonferroni correction. *P* values of <0.05 were considered significant. Each test was performed in triplicate and in three independent experiments. For confocal microscopy two experiments were performed.

## Results

### *T. rubrum* ATCC MYA 4438 Strain Forms Stronger Biofilms Than the BR1A Clinical Strain

**Figure 1:**
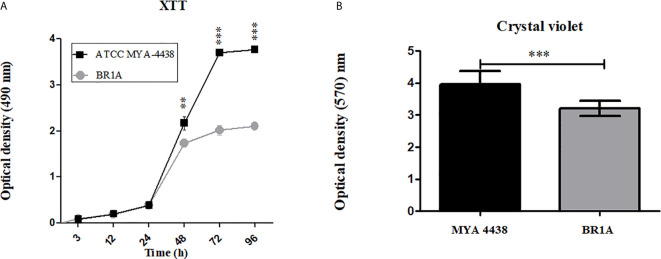
*T. rubrum* ATCC MYA-4438 strain forms stronger biofilms than the clinical isolate BR1A strain. **(A)** Kinetics of *T. rubrum* biofilm formation in 96-well plates determined by XTT reduction assay. **(B)** Quantification of the biomass of mature *T. rubrum* biofilms after 72 h incubation was performed using crystal violet staining. For panels a and b, each time point denotes the average of 3 independent wells. Error bars indicate standard deviations. p values (**p **<** 0.01; ***p **<** 0.001) were calculated by student**’**s *t* test analysis.

The kinetics of biofilm formation by the *T. rubrum* strains ATCC MYA 4438 and BR1A was quantified by the XTT reduction assay (**Figure 1A**). The initial changes in metabolic activities occurred after 3 h of incubation (pre-adhesion period). Both strains experienced similar slow increases in their metabolic activities over the first 24 h of incubation, followed by a substantial increase thereafter. At 48 h and subsequent time points, *T. rubrum* ATCC MYA 4438 produced biofilms that were significantly more metabolically active compared to the BR1A strain (p <0.01). Also, BR1A reached a metabolic activity plateau after 48 h whereas ATCC MYA 4438 reached a plateau after 72 h. For both strains, biofilm maturation or establishment was considered after 72 h incubation. We quantified the biomass of each mature biofilm by crystal violet staining (**Figure 1B**). Biofilms formed by the *T. rubrum* ATCC MYA 4438 strain of showed significantly higher biomass than the clinical strain BR1A biofilms after 72 h (p <0.01). These findings indicate that *T. rubrum* ATCC MYA 4438 strain forms more robust biofilms than the BR1A clinical strain.

### NO-np Reduced the Metabolic Activities of Planktonic Cells of Both Strains and of the Preformed Biofilm of the Clinical Strain BR1A

To evaluate the susceptibility of planktonic cells and established biofilms of *T. rubrum* to NO-np and antifungal drugs, their metabolic activities were quantified using the XTT reduction assay. NO-np reduced the metabolic activities of planktonic cells at a concentration of 20 mg/mL in both strains, and of the preformed biofilm by the clinical strain BR1A at a concentration of 40 mg/mL (**Table 1**). There was a slight reduction in the metabolic activities of the biofilm formed by the ATCC MYA 4438 strain after treatment with NO-np (~20%; data not shown), but not enough to be considered an inhibition according to the pre-established parameters (SMIC> 40 mg/mL). The control np were not active for any phenotype (MIC and SMIC> 40 mg/mL), confirming the action of NO. Regarding antifungal drugs, FCZ, TRB and EFCZ reduced the metabolic activities of planktonic cells at low concentrations (0.000125 - 0.016 mg/mL), but did not reduce the metabolic activities of preformed biofilms at the higher concentrations tested. Thus, NO-np significantly reduced the metabolic activity of biofilms but at higher concentrations than the antifungal drugs. Similarly, *T. rubrum* biofilms are more resistant to NO-np and antifungal drugs than planktonic cells.

**Table 1:** Susceptibility of *T. rubrum* ATCC MYA 4438 or BR1A planktonic cells or biofilms to nitric oxide nanoparticles (NO-np), control nanoparticles (np), fluconazole (FCZ), terbinafine (TRB), or efinaconazole (EFCZ).

Fungi/np/drugs	PLANKTONIC CELLS		BIOFILMS	
	*T. rubrum* ATCC MYA 4438 (MIC_50_ mg/mL)	*T. rubrum* BR1A (MIC_50_ mg/mL)	*T. rubrum* ATCC MYA 4438 (SMIC_50_ mg/mL)	*T. rubrum* BR1A (SMIC_50_ mg/mL)
**NO-np**	20	20	>40	40
**np**	–	–	–	–
**Fluconazole**	0.008	0.016	>0.512	>0.512
**Terbinafine**	0.00003	0.000125	> 0.032	>0.032
**Efinaconazole**	0.000125	0.0006	>0.320	>0.320

MIC_50_, minimum inhibitory concentration capable of reducing at least 50% of the metabolic activities of planktonic cells; SMIC_50_, sessile minimum inhibitory concentration capable of reducing at least 50% of the metabolic activities of mature biofilms; mg/mL, milligrams per milliliters).

### NO-np Damages the Architecture of ATCC MYA-4438 and BR1A Biofilms

**Figure 2:**
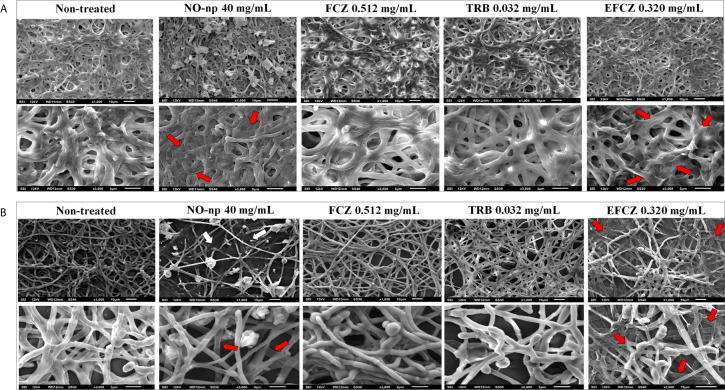
Scanning electron microscopy showing the architecture of biofilms for **(A)** *T. rubrum* ATCC MYA-4438 and **(B)** BR1A strains. Different magnifications (upper panel; 1000 and lower panel; 3000X) are shown for untreated biofilms and those treated with NO-np 40 mg/mL, fluconazole (FCZ) 0.512 mg/mL, terbinafine (TRB) 0.032 mg/mL, or efinaconazole (EFCZ) 0.320 mg/mL. Red arrows denote collapse of the hyphal walls, while white arrows denote reduced hyphal density.

The structure of non-treated and NO-np or antifungal drug treated biofilms were visualized by SEM. The biofilms formed by the ATCC MYA-4438 strain (**Figure 2A**) were more robust, dense, and compact than those formed by the BR1A strain (**Figure 2B**), validating the results of the colorimetric tests. Biofilms from both strains showed considerable damage after incubation with NO-np 40 mg/mL (**Figures 2A, B**) and EFCZ 0.320 mg/mL (**Figures 2A, B**). These findings are interesting in light of the XTT reduction assay results, which indicated high metabolic activity after these treatments. Collapse of the hyphal walls were observed, mainly in the biofilms formed by the ATCC MYA-4438 strain. However, a greater destructive effect was observed in biofilms formed by the BR1A strain, resulting in reduced fungal cell density and hyphal density. There was no significant structural damage to biofilms treated with either FCZ (0.512 mg/mL) or TRB (0.032 mg/mL). (**Figures 2A, B**). These images confirmed that NO-np is more effective in damaging and eradicating *T. rubrum* biofilms than commonly used antifungal drugs.

### NO-np Reduces the Thickness of *T. rubrum* Biofilms to Similar Extent as EFCZ

**Figure 3:**
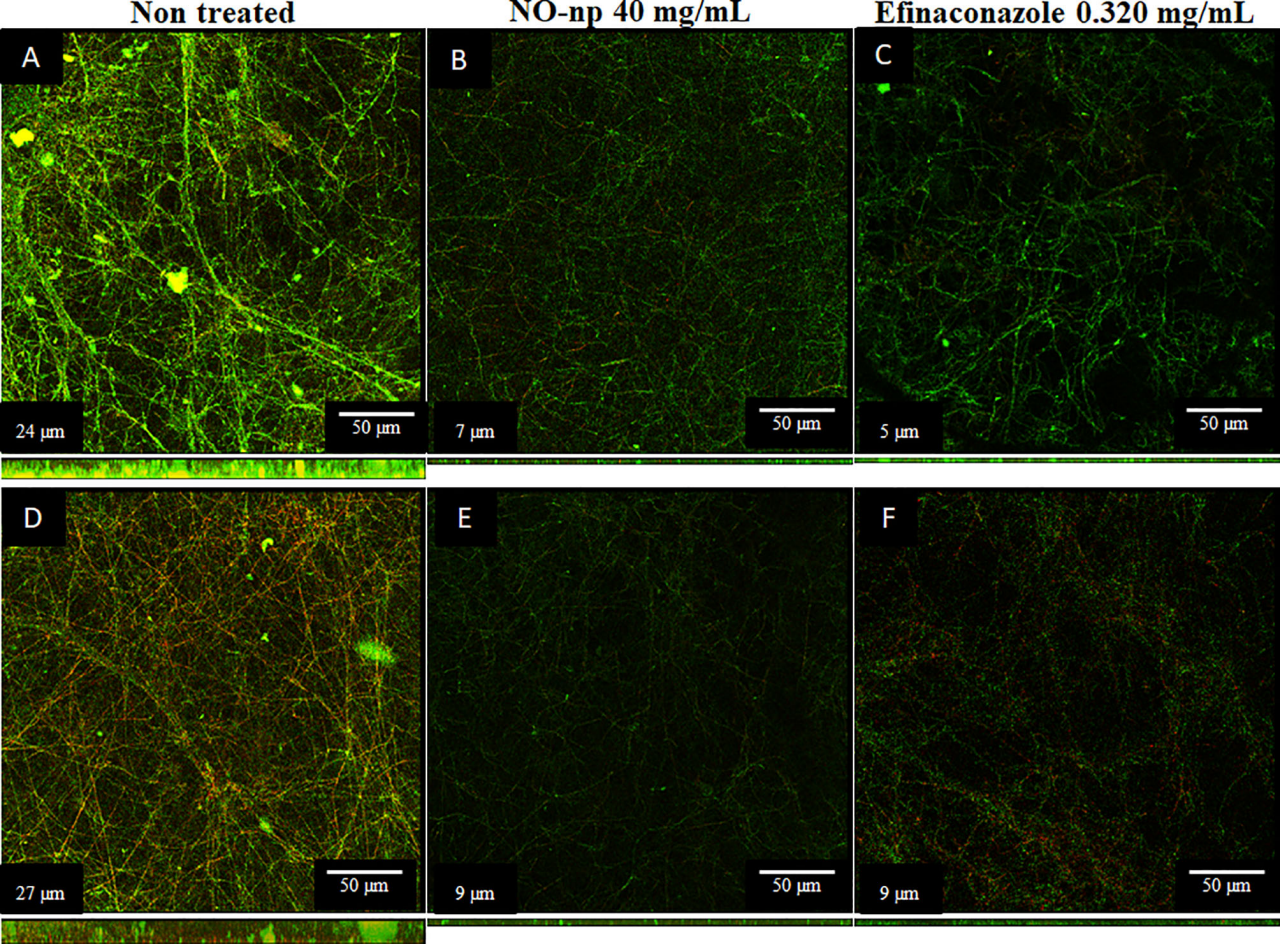
Confocal microscopy images of mature *T. rubrum* biofilms formed on glass-bottom plates for 72 h at 37°C **(A, D)** and treated with NO-np **(B, E)** or EFCZ **(C, F)**. Orthogonal images of mature *T. rubrum* biofilms showed metabolically active (red, FUN-1-stained) cells embedded in the polysaccharide extracellular material (green, ConA), while the yellow-brownish areas represent metabolically inactive or nonviable cells. Images were obtained after 72 h of exposure of the fungal cells to 40 mg/mL of NO-np or 0.32 mg/mL of EFCZ, and the images were compared with those of biofilms incubated in presence of RPMI. The pictures were taken at a magnification of ×63. Bars, 50 μm. The thickness of the fungal biofilms grown under these conditions was measured by *z*-stack reconstruction. The results are representative of those of two experiments.

Confocal microscopy was performed to test the activity of NO-np and EFCZ on the structure and metabolism of *T. rubrum* biofilms. Orthogonal microscopic sections show regions with high metabolic activity (FUN-1; red); regions in green that correspond to the cell walls of hyphae and polymeric extracellular material (Con-A) and in yellow-brownish the metabolically inactive regions. Non-treated *T. rubrum* biofilms show extensive hyphal distribution throughout the field and evident high metabolic activity **(** **Figures 3A, D** **)**. Although both *T. rubrum* strains showed similar biofilm thickness, ATCC MYA-4438 strain biofilms **(** **Figure 3A** **)** consisted of high density and thick hyphal threads whereas BR1A strain biofilms **(** **Figure 3D** **)** exhibited low density and thin hyphae. Both, NO-np **(** **Figures 3B** **–E** **)** and EFCZ **(** **Figures 3C–F**), demonstrated comparable activity against fungal biofilms. Fungi treated by either NO-np or EFCZ demonstrated a reduction in hyphal accumulation and extracellular matrix, leading to a reduction in the thickness of biofilms (**Figures 3B, C, E, F**). These findings show that NO-np is similarly effective in killing *T. rubrum* biofilms in comparison with a conventional antifungal drug.

## Discussion

In this work, the *in vitro* biofilm formation by two strains of *T. rubrum* was characterized using the colorimetric assays XTT and crystal violet as well as by SEM. These methods are widely used and complementary in the study of biofilms. The three tested methodologies demonstrated that the MYA-4438 strain formed more robust biofilms after 72 h than the clinical strain BR1A. Previous studies demonstrated that biofilms by dermatophytes *in vitro* mature after 72 h, although variations between different strains have also been reported (Costa-Orlandi et al., 2014; Brilhante et al., 2017; Chen et al., 2019; Costa-Orlandi et al., 2020; Garcia et al., 2020).

The ability of NO-np to inhibit biofilm formation when co-incubated with planktonic cells and to damage established biofilms was evaluated and compared to FCZ, TRB and EFCZ. Planktonic cells were more susceptible to treatment with NO-np and antifungal drugs compared to biofilm-derived cells. The increased resistance of fungal biofilms to antimicrobials is well reported in the literature (Di Bonaventura et al., 2006; Martinez and Casadevall, 2006; Mowat et al., 2007; Gupta and Foley, 2019). The images of the biofilm structures after treatment with FCZ and TRB corroborated those found by the XTT reduction assay and showed the resistance of these complex communities to two of the most used drugs for the treatment of dermatophytoses. The resistance of dermatophyte biofilms to TRB corroborates previous findings (Toukabri et al., 2018; Garcia et al., 2020). In contrast, SEM images showed severe damage to the biofilm structure of both strains after treatment with NO-np or EFCZ, contradicting the findings of the quantification of metabolic activities. We hypothesize that the difference is due to the limitations of the XTT reduction assay. In this regard, Chandra et al. (2008) previously suggested that there may not be a linear relationship between the number of microorganisms and the colorimetric signal. In addition, other studies report conflicting results with cell viability in studies using XTT and MTT in tests with nanoparticles (Wang et al., 2011; Costa-Orlandi et al., 2020). Both reagents, when in contact with some nanoscale particles, may underestimate toxicity and overestimate cell viability.

Although the SEM images revealed biofilm damage after treatment with NO-np or EFCZ, their visualization is limited. Therefore, confocal microscopy was used to further corroborate the effects of each treatment on these communities. In addition to causing a reduction in the biofilm thickness for both strains, these treatments also reduced the metabolic activity and extracellular matrix distribution of the fungal biofilms. The NO-np used in the present work has broad spectrum applications against fungi and a variety of gram positive and negative bacteria, in addition to the immunomodulatory effects and tissue regeneration functions (Martinez et al., 2009; Mihu et al., 2010; Macherla et al., 2012; Sanchez et al., 2012; Mordorski et al., 2017; Costa-Orlandi et al., 2018). Its anti-dermatophyte activity has been proven both in *in vitro* tests on planktonic cells and in a murine model of dermal infection (Mordorski et al., 2017; Costa-Orlandi et al., 2018). For dermatophytes, NO exerts a fungistatic action and promotes an increase in intracellular vacuoles and vesicles (Mordorski et al., 2017). NO can act through multiple non-specific mechanisms, which include damage to genetic material, lipid peroxidation, inactivation of enzymes, and macrophage upregulation (Tümer et al., 2007; Qin et al., 2015; Mordorski et al., 2017). This target multiplicity makes it difficult for microorganisms to develop resistance (Qin et al., 2015; Costa-Orlandi et al., 2018). The commercially available drugs used in this work have a common target, which is the main component of the fungal membrane, ergosterol. FCZ and EFCZ interfere with the synthesis of ergosterol *via* cytochrome P450, inhibiting the enzyme 14-alpha-demethylase, involved in the conversion of lanosterol to ergosterol, resulting in alteration of the fungal cell membrane and growth inhibition (Patel and Dhillon, 2013; Scorzoni et al., 2017). TRB targets the enzyme squalene epoxidase, which is involved in the first steps of ergosterol biosynthesis. This inhibition causes the accumulation of squalene and the absence of ergosterol precursors, resulting in cell death (Scorzoni et al., 2017). These mechanisms can complement each other and there is already evidence that, in combination with EFCZ, NO-np showed a synergistic effect against *T. rubrum*, suggesting a promising alternative for maintaining activity and reducing treatment costs (Costa-Orlandi et al., 2018).

It is important to note that NO-np do not show significant toxicity in human cell lines, in a zebrafish model or in topical, intraperitoneal and intravenous applications in animals (Friedman et al., 2008; Cabrales et al., 2010; Han et al., 2012; Mordorski et al., 2017; Costa-Orlandi et al., 2018), which makes them a potential viable, safe and effective topical alternative for the treatment of biofilms complicating onychomycosis.

## Conclusion

We demonstrated the effectiveness of a NO-np against *T. rubrum* biofilms, and showed that the activity was comparable to that observed with EFCZ. In addition, we also confirmed the resistance of the biofilms to two drugs widely used in the treatment of dermatophytosis, TRB and FCZ, which may contribute to the prolonged treatment requirements and the common recurrences observed in individuals previously affected with in this mycosis. Studies regarding the anti-biofilm effect of NO-np should be further investigated and considered, since this phenotype is prevalent in onychomycosis and is partly related to the low success rates of healing for this clinical manifestation. Also, these findings support the concept that biofilms should be considered when developing and testing new drugs for the treatment of dermatophytosis.

## Data Availability Statement

The original contributions presented in the study are included in the article/supplementary material. Further inquiries can be directed to the corresponding authors.

## Author Contributions 

CC-O, MM-G, and JN conceived and designed the study. CC-O and LM performed the experiments. CC-O and NB analyzed the data and wrote the manuscript. AF and JF are co-inventors of the nitric oxide releasing nanoparticles used in this study. All authors contributed to the article and approved the submitted version.

## Funding

This work was supported in part by Coordenação de Aperfeiçoamento de Pessoal de Nível Superior (CAPES) [Finance code 001; 99999.007910/2014-02 (CC-O)]; Programa de Apoio ao Desenvolvimento Científico (PADC) da Faculdade de Ciências Farmacêuticas da UNESP; Fundação de Amparo à Pesquisa do Estado de São Paulo-FAPESP [2017/18388-6 (CC-O), 2018/02785-9 (MM-G), 2019/22188-8 (NB)] and Conselho Nacional de Desenvolvimento Científico e Tecnológico (CNPq) [310524/2018-1 (MM-G)]. LM was supported by the National Institute of Allergy and Infectious Diseases (NIAID award # R01AI145559) of the US National Institutes of Health (NIH).

## Conflict of Interest

The authors declare that the research was conducted in the absence of any commercial or financial relationships that could be construed as a potential conflict of interest.
